# Dissecting single-cell genomes through the clonal organoid technique

**DOI:** 10.1038/s12276-021-00680-1

**Published:** 2021-10-18

**Authors:** Jeonghwan Youk, Hyun Woo Kwon, Ryul Kim, Young Seok Ju

**Affiliations:** 1 Graduate School of Medical Science and Engineering, Korea Advanced Institute of Science and Technology (KAIST), Daejeon, 34141 Republic of Korea; 2 GENOME INSIGHT Inc, Daejeon, 34051 Republic of Korea; 3 Department of Nuclear Medicine, Korea University College of Medicine, Seoul, 02841 Republic of Korea

**Keywords:** Genomic analysis, Genomics, Adult stem cells, Genetic techniques

## Abstract

The revolution in genome sequencing technologies has enabled the comprehensive detection of genomic variations in human cells, including inherited germline polymorphisms, de novo mutations, and postzygotic mutations. When these technologies are combined with techniques for isolating and expanding single-cell DNA, the landscape of somatic mosaicism in an individual body can be systematically revealed at a single-cell resolution. Here, we summarize three strategies (whole-genome amplification, microdissection of clonal patches in the tissue, and in vitro clonal expansion of single cells) that are currently applied for single-cell mutational analyses. Among these approaches, in vitro clonal expansion, particularly via adult stem cell-derived organoid culture technologies, yields the most sensitive and precise catalog of somatic mutations in single cells. Moreover, because it produces living mutant cells, downstream validation experiments and multiomics profiling are possible. Through the synergistic combination of organoid culture and genome sequencing, researchers can track genome changes at a single-cell resolution, which will lead to new discoveries that were previously impossible.

## Genomics: Culturing mini-organs improves the accuracy of single-cell sequencing

The combination of three-dimensional tissue culturing and the latest DNA sequencing methods is allowing researchers to track genetic changes in single cells in ways that were previously impossible. A team led by Young Seok Ju from the Korea Advanced Institute of Science and Technology in Daejeon, South Korea, review some common techniques by which biologists catalog mutations at single-cell resolution. The most precise and informative, they suggest, involves first growing cells in the laboratory in three-dimensional aggregates known as organoids, which mimic tissue structures in the body, before running genome-sequencing assays. Although the tissue culturing adds extra work, the authors maintain that the additional biological material that organoids provide yields the most accurate mutational profile of the cells under investigation, which is of value in many research settings.

## Introduction

Over the past decade, advances in DNA sequencing technology have enabled us to explore entire genome sequences in faster and cheaper ways^1^. As of 2020, ~30-fold human whole-genome sequences could be produced in less than a week with a cost of <$1,000^1,2^. With this technology, researchers are able to access the vast majority of, if not all, genetic variations in an individual for the first time^3,4^. Because these variations are the most important element underlying the phenotypic diversity of human individuals, their sensitive and accurate detection is critical for precision health care. More fundamentally, mutations are cumulative records of DNA damage and repair processes through the germline and somatic lineages^5^. Therefore, the burden and patterns of genomic variations provide deep insights into the mutational processes operative over time^6^.

**Fig. 1:**
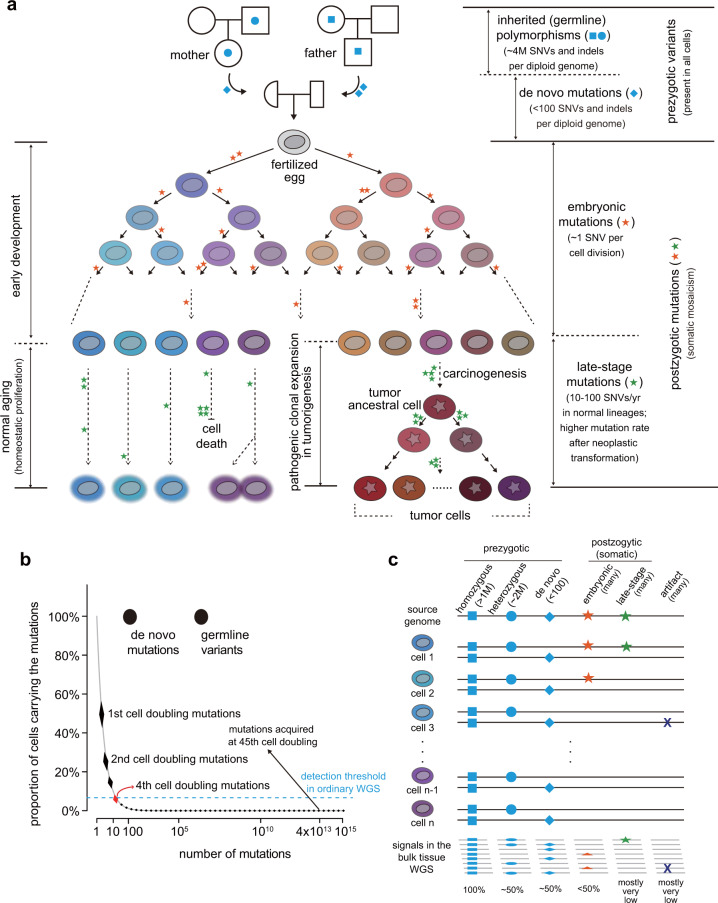
The origin of genomic variants in human cells and their characteristics. **a** The origin of genomic mutations together with the cellular phylogeny of an individual’s development. Four different origins (inherited, de novo, and embryonic and late stages) are represented. **b** The characteristics of genomic variants according to their origin. The mutation number per cell (*x*-axis) and the proportion of mutant cells in an individual’s body (*y*-axis) are illustrated. For postzygotic mutations, we assume that the endogenous mutation rate is 1 mutation per cell per cell doubling. The threshold of mutation detection (6.6% of the cell fraction) is calculated with 30-fold whole-genome sequencing. **c** The signal intensity of genomic variants in ordinary bulk-tissue whole-genome sequencing according to the origin of the genomic variants. WGS, whole-genome sequencing.

Humans are multicellular organisms. An adult body consists of 40 trillion somatic cells developing from a single common ancestral cell, the fertilized egg^7^. Through a series of mitotic cell divisions, the single-cell organizes a complex multicellular system during the developmental stage. After full development and growth, many tissues are maintained via the homeostatic proliferation of adult stem cells that compensate for the loss of differentiated cells^8^. It is generally assumed that the genetic constitution of all the somatic cells of an individual is virtually identical and uniform. Indeed, all cells in an individual harbor 3–4 million germline polymorphisms inherited from both parents^9^ as well as 50–100 de novo mutations acquired during the formation of germ cells^10,11^ (Fig. 1a, b). However, these are not all the genetic variants present in the cellular pool of an individual. Postzygotic mutations, also known as somatic mutations, can accumulate during every mitosis from the first cell division in life onward^12^ (Fig. 1a, b). The concept of accumulating genomic changes was first proposed in 1959^13^, but the incidence and underlying molecular mechanisms across cellular lineages have been poorly characterized^14^, primarily due to the lack of techniques for the sensitive discovery of somatic mutations restricted to single cells. In theory, the acquisition of somatic mutations occurs via independent events in each cell caused by exposure to external mutagens or endogenous mutagenic processes, such as spontaneous methylcytosine deamination, errors in DNA replication, and the misregulation of APOBEC enzymes (apolipoprotein B mRNA editing enzyme or catalytic polypeptide-like enzymes)^5^. These mutations are known to cause various human diseases, including not only cancers but also neurodevelopmental and immune-related disorders^5,15–19^.

It is estimated that roughly 0.1–10 mutations can be acquired during every mitosis of human cells^10,12,14,20^. If ~1 somatic mutation arises in each of the two daughter cells of a fertilized egg, the adult body will acquire two mutations overall from the first cell division. Unlike inherited and de novo mutations, these acquired mutations are not shared by all cells in an individual body. Instead, if the two earliest cells contribute more or less equally to an adult body, each of the two mutations will be carried by one of the two cellular subsets, each constituting ~50% of the cells in an individual body (Fig. 1b). Assuming a similar mutation rate in later cell generations, four and eight mutations will arise in the four- and eight-cell stages, respectively, and the mutations will be harbored by ~25% and ~12.5% of the adult cell pool. In this scenario of the continuous accumulation of one mutation per cell per cell division, the 40 trillion cells in an individual body collectively acquire 40 trillion mutations from the most recent cell division in each somatic lineage, which far outnumbers the total bases of the human genome (~3 billion nucleotides) (Fig. 1b). Through continuous homeostatic cellular proliferation over an individual’s lifetime, the level of somatic mosaicism increases, and the genome sequences of cells become more heterogeneous.

Until recently, somatic mutations were cataloged mostly from cancer tissues^21^. Neoplastic cells in a cancer tissue are formed by clonal expansion from the most recent common ancestral (MRCA) cell^22^; thus, all the somatic mutations carried in the MRCA cell are shared by all cancer cells (Fig. 1a) if we ignore the small possibility of deletions involving previously mutated loci during tumor progression. These shared mutations can be detected by the conventional 30-fold whole-genome sequencing (WGS) of tumor bulk tissues. This technical possibility enabled an international effort to systematically catalog somatic mutations from >10,000 human cancers in the last decade^23,24^. By contrast, polyclonal nonneoplastic tissues are much more challenging for genome analyses, as most mutations are confined to single cells or to a very small fraction of cells in the tissue; thus, weak signals from the mutation are difficult to distinguish from ubiquitous technical errors arising from the sequencing process^25^ (Fig. 1c). The deep genome sequencing of polyclonal bulk tissues and duplex DNA sequencing^26–28^ are applicable for exploring somatic mutations present in a given tissue, but these mutations lack sensitivity and a single-cell resolution. Nevertheless, multiple lines of evidence now suggest that the progressive accumulation of genomic aberrations is a common feature of apparently healthy cells as they age and is the source of human diseases^26,29,30^. Their functional impacts and association with human diseases have not been explored due to the lack of technology facilitating systematic discoveries. With the reduction of sequencing costs, exploring somatic mosaicism by sequencing multiple genomes from the same individual has become affordable.

Recently, organoid technologies that facilitate long-term clonal expansion of stem cells were developed^31,32^. Organoid technologies allow adult tissue stem cells to expand and self-organize into an organotypic 3D tissue architecture. Because organoids recapitulate the cellular physiology of in vivo tissues, these models have been used to study the pathogenesis of many diseases, such as infections, genetic disorders, and cancers. Additionally, as we will further discuss below, organoids provide great opportunities for the study of somatic mutations in single cells without error-prone whole-genome amplification.

In this review article, we provide an overview of three approaches currently being used for the detection of somatic mutations accumulated in single cells. The details of each approach, along with their advantages and limitations, are discussed. The combination of organoid and genome sequencing technologies yields the most sensitive and precise mutation catalog, at the expense of intensive labor requirements. We predict that the landscape of somatic mosaicism among the various cell types of young and aged individuals or healthy and diseased individuals will be explored via the synergistic integration of these techniques.

## Main text

### Amount of DNA required for genome sequencing

As of 2020, Illumina platforms, such as NovaSeq, were being dominantly applied for population-scale genome sequencing. The accuracy and efficiency of DNA library preparation have been greatly improved over the last decade. Current standard library preparation kits require ~100 ng of genomic DNA as the input material. Specialized low-input DNA library kits can be used with ~1 ng of genomic DNA^33^, although the quality of genome sequencing is usually compromised, with lower library complexity and higher levels of GC bias and sequencing error rates^34^. In principle, genomic DNA from a single cell should be exclusively employed to detect somatic mutations restricted to a single cell. However, because a human diploid cell contains only ~0.006 ng (6 pg) of genomic DNA^35^, the direct sequencing of a single genome is physically not possible. To overcome this challenge, researchers have amplified single genomes through several strategies.

### Whole-genome amplification

**Fig. 2:**
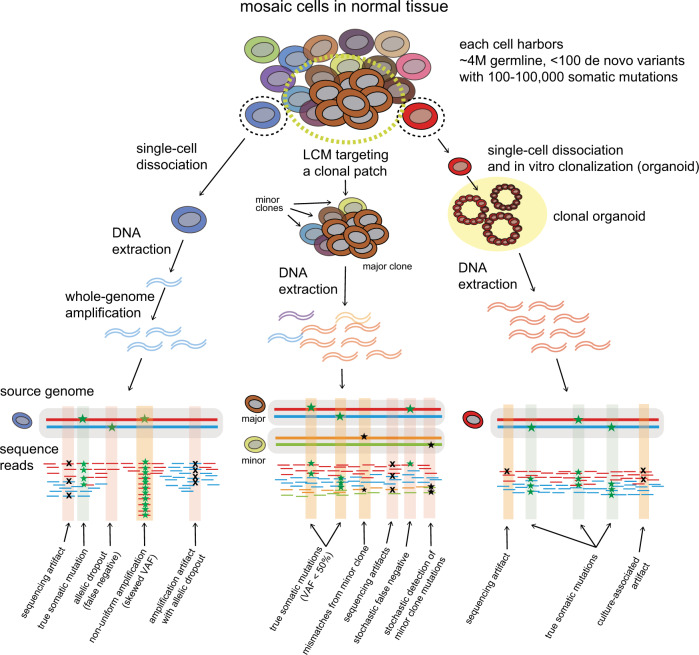
Three strategies for detecting genome-wide mutations in single cells. Three different strategies that can be applicable for detecting single-cell mutations are illustrated with brief experimental steps. Expected outcomes from each of the strategies are shown below with summaries of source genomes and sequence reads produced from the source. True, borderline, and false findings are highlighted with green, orange, and red boxes, respectively. LCM laser-capture microdissection.

A single DNA copy can be exponentially duplicated using in vitro amplification methods, such as polymerase chain reaction (PCR). Using the whole-genome amplification (WGA) technique, picograms of single-cell genomic DNA can be amplified to the microgram scale^36^ (Fig. 2). In the 1980s−1990s, early PCR-based WGA strategies were designed by the ligation of specific primers to sheared DNA fragments (linker-adapter PCR)^37^ or by targeting specific primers to repetitive sequences in the genome^38^. More efficient techniques then followed, including (1) degenerate oligonucleotide-primed PCR (DOP-PCR), which is an exponential PCR method with random primers^39^; (2) multiple displacement amplification (MDA), which uses a highly processive and strand-displacing phi29 DNA polymerase to exponentially amplify single-stranded DNA in isothermal conditions^40,41^; and (3) multiple annealing and looping-based amplification cycles (MALBAC), which leads to nonspecific but quasi-linear DNA amplification by protecting the amplification products in a loop structure that is not further amplified^42^. More recently, the linear amplification via transposon insertion (LIANTI) method was developed, in which genomic DNA is transcribed multiple times in vitro, and the expressed RNAs are sequenced^43^. These techniques each have their own pros and cons, as illustrated in previous review articles^36,44^.

Although WGA approaches seem straightforward for single-genome sequencing, they create a large number of amplification biases and errors, including artificial false mutations, allelic dropout, and preferential amplification of one of the two alleles (Fig. 2). For example, in single-nucleotide variant detection experiments, DOP-PCR, MDA, and MALBAC produce 1–40 artifact mutations per 100 kb^43^. On a genome-wide scale, these processes can generate 30,000–1,200,000 artificial point mutations, which is a much higher number than the number of true somatic mutations in single cells (usually <10,000 mutations)^45^. Despite the existence of many downstream bioinformatics strategies, it is quite challenging to filter out the excessive number of experimental artifacts^46^. In addition, due to frequent allelic dropout, a high fraction of true variants (21–76%) can be lost during the WGA process^47^. Moreover, amplification noise interferes with the accurate detection of DNA copy number changes^36^, particularly when the length of amplifications/deletions is short (<100 kb), so true somatic structural variations are difficult to detect.

Despite these limitations, WGA-based single-genome sequencing has been applied to explore single-genome mutations, particularly in nonproliferating cells such as neurons^48–50^. Although sequences are sometimes heavily contaminated by errors during WGA steps, some features of these genomes, such as the mutational burden and functional mutations shared by multiple cells, have been explored by using these techniques^46^.

### Microdissection of clonal patches

Similar to cancer tissues, some adult stem cells can proliferate more quickly than neighboring stem cells in normal tissues, generating a ‘clonal patch’. For instance, in the normal intestinal crypt, a few equivalent stem cells continuously compete for residence^51^ (Fig. 2). Among these cells, one clone may produce more progeny in a given time resulting from either a stochastic mechanism or acquired competence induced by somatic genetic/epigenetic alterations^52^, or both. The generation of phenotypic diversity through the continuous acquisition of new alterations and the natural selection of more effectively proliferating clones is the main tumorigenic process in normal tissues^5^.

Under these circumstances, the microscopic structure is continuously converted into single-clone compartments over time. The territory of a stem cell can spread even further through rounds of dynamic fission and fusion of its crypts^53^. Although the frequency in various tissue types is uncertain, clonal patches are known to form in many tissue types, such as the liver^54^, uterus^52^, placenta^55^, and other pan-body tissues^56^. As in cancer tissues, the cells in a clonal patch share all somatic mutations acquired in the genome of the MRCA cell. Using specialized technologies such as laser capture microdissection (LCM), these patches can be physically separated, followed by WGS without error-prone WGA^33^. In this way, somatic mutations in normal tissues can be efficiently detected.

In practice, microdissected tissues are not always single clones but usually include multiple clones^56^, either because the size of the clonal structure is smaller than the dissected structure or because the excised tissue overlaps the boundaries between multiple clonal patches. Clonality in excised tissue can be inferred from genome sequences using the number of discovered somatic mutations and their variant allele fractions (VAFs)^56^. Monoclonal patches show a substantial number of somatic mutations, with a VAF peak of ~50%. Oligoclonal patches provide many somatic mutations with a VAF distribution substantially lower than 50% according to the proportion of the leading clone. Polyclonal patches, composed of many clones, none of which achieve clonal dominance, yield very few mutations.

Despite these limitations, LCM has shown the ability to scalably detect somatic mutations in nonneoplastic cells. For instance, Lee-Six et al. dissected 571 colonic crypts using LCM followed by WGS^57^. The VAF distribution clearly suggested that ~90% of the crypts were clonalized at a certain time in the life cycle, estimated to be 5.5 years earlier than the age at dissection. The mutation rate of adult stem cells in crypts was 43.6 somatic base substitutions per year, and ~1% of the adult stem cells in the apparently normal colorectal crypts harbored known cancer driver mutations, such as *AXIN2* and *PIK3CA*. Morphologically normal bladder^58,59^, liver^54^, endometrium^52^, and pan-body tissues of human individuals^56^ have been explored by similar techniques.

### Clonalization of single cells

Mammalian cells accurately duplicate their genomes during every cell division using high-fidelity DNA polymerase in combination with DNA repair mechanisms. Relative to the error-prone ex vivo WGA approach mentioned above, the accuracy of intracellular genome duplication is considerably higher (at least 1,000 times)^20,43,60^. Therefore, the in vitro clonal expansion of single cells to hundreds of cells may be an option for amplifying single-cell DNA for WGS (Fig. 2).

To implement these strategies, researchers have applied various cell culture techniques, such as classical 2D culture, induced pluripotent stem cell (iPSC) culture, and organoid culture technologies. Some cell types, such as hematopoietic stem cells^61^, bronchial epithelial cells^62^, and fibroblasts^63^, can be successfully clonalized using primary 2D culture techniques. However, unlike cancer cell lines, most primary human cells are difficult to expand in vitro using the classical technique, particularly when a single cell is isolated. Alternatively, the iPSC technique has the advantages of fast growth and ease of cloning. Using this approach, somatic mutations in single cells due to exposure to carcinogens were systematically explored^64^. However, the conditions required to induce dedifferentiation are not close to physiological conditions, and this step thus constitutes a strict selective bottleneck that may be permissive to a specific group of cells. For example, the efficiency of iPSC establishment from differentiated cells is ~1% under the best conditions^65,66^.

For the clonal expansion of single cells in various human tissues, organoid technologies have many advantages over cell culture methods. Most importantly, various human cell types can be physiologically expanded using this technology. From the first report involving the small intestine^67^, primary culture conditions for at least 15 tissue stem cells^31^, including colon, pancreas, liver, prostate, stomach, fallopian tube, uterus, placenta, skin, breast, and lung cells, have been thoroughly established^68–82^. By applying these techniques, adult single stem cells can be rapidly expanded in chemically defined media with high efficiency. Feeder cell-free culture conditions prevent potential DNA contamination, which could cause inaccurate genome analysis. Because organoid culture conditions mimic physiological tissue environments, the long-term culture of a clone (>1 year) is possible while maintaining genomic stability^83^.

### Pros and cons of the approaches

Each approach has its own advantages and drawbacks when applied to single-genome analyses (Table 1). WGA is technically simple, straightforward and applicable to nondividing cells but suffers from a large number of false-positive and false-negative calls. The quality of WGA genome sequences should be thoroughly investigated. The compromised genome coverage (allelic dropout) and levels of sequencing artifacts under this approach can be partially explored with downstream bioinformatic techniques, such as analyses of the genome-wide variation of sequencing read depth, the proportion of discordant paired-end reads, and the presence of error signatures^84^. The microdissection technique is accurate and easy to scale up but requires preclonalized anatomical structures, which are not always present in human tissues. As briefly mentioned above, the clonality of patches can be investigated according to the distribution of VAFs in somatic mutations^56^. Genomes in polyclonal patches cannot be further investigated due to the loss of sensitivity in the mutation detection, as mentioned above. Compared with these two approaches, in vitro clonalization requires more complicated steps, such as single-cell dissociation and proliferation, and is thus more expensive, labor-intensive, and time-consuming. In particular, the single-cell dissociation step in the organoid culture method is technically sophisticated, requiring extensive trypsinization and FACS sorting^85^. Single-cell proliferation is not rapid in certain cell types even in organoid cultures, as observed for alveolar type 2 cells of the lung (3–4 doublings every 2 weeks)^79^. Moreover, clonalization is limited to dividing cells, and if the process is not carefully controlled, culture-associated mutations can contaminate the genome^86^. As in WGA and microdissection, these features of the clonalization technique can be confirmed by downstream bioinformatics QC steps^85^. Despite these limitations, clonal expansion is a unique way to sensitively capture entire genomic loci from physically isolated single cells. Using this approach, the most accurate and sensitive catalog of somatic mutations, including base substitutions, indels, copy number changes, and structural variations, can be obtained. Moreover, this approach produces viable cells that can be used for further experimental analyses to validate the insights obtained from genomic analyses^87^. For example, the transcriptomes and epigenomes of a specific clone, as well as phenotypes of the cells, such as proliferation rate, tumor-forming ability, and resistance to specific chemicals, can be further explored at a single-clone resolution with the established cells.

**Table 1:** Comparison of the three approaches for investigating single-cell genomes.

	Whole-genome amplification	Laser capture microdissection	In vitro clonalization
Sensitivity of somatic mutation detection	++	++	+++
Amplification artifact (false positive)	+++	++	+
Culture-associated artifact (false positive)	−	−	+
Allelic dropout (false negative)	+++	−	−
Dependency on tissue clonality	−	+++	−
Opportunity for further experiments	−	+	+++
Applicability to nonproliferating cells	+++	+++	−
Spatial information	−	+++	−
Time	+	++	+++
Cost	+	++	+++
Technical challenges	+	++	+++

Here, we compared three different technologies that are applicable for detecting mutations in single cells. Relative scores are represented as very high (+++), high (++), medium (+) and low (−).

### Applications of the clonal organoid technique

#### Cellular phylogenies in early embryonic development

**Fig. 3:**
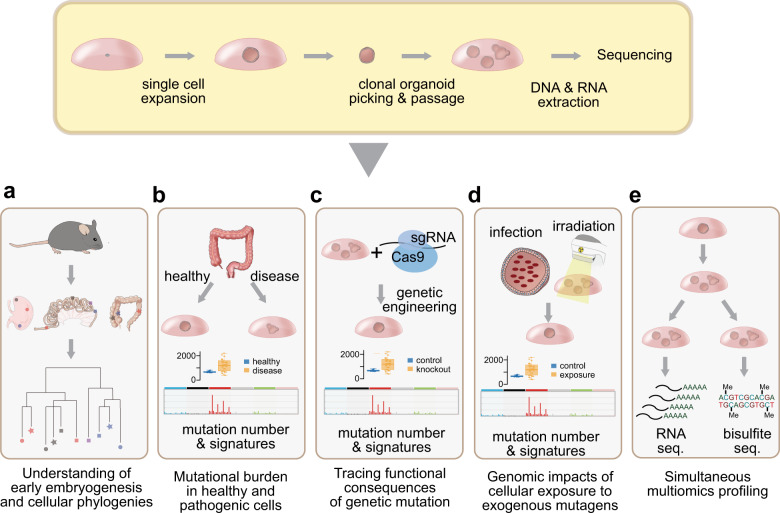
Applications of the clonal organoid technique. **a** By reconstructing cellular phylogenies using somatic mutations as barcodes, clonal dynamics in early development can be traced. **b** The genome-wide mutational burden and signatures can be investigated in normal and diseased cells when clonal organoids are derived from healthy and diseased tissues. **c** The functional impact of a specific gene mutation can be investigated via the genetic engineering of organoids followed by sequencing. **d** By exposing organoids to carcinogens, their mutagenic impacts can be accurately assessed. **e** Organoid technologies enable multiomics profiling because a large number of progeny cells, which share similar genomic and phenotypic backgrounds, can be produced.

The WGS of clonalized organoids was first applied to reconstruct the early developmental phylogenies of many somatic cells collected from various murine tissues (Fig. 3a)^88^. As the mutations arising in an embryonic cell are inherited by its descendant cells, these mutations can be used as cellular barcodes to reveal their early founder cells. Behjati and colleagues established 25 clonal organoids from stomach, small intestine, colon, and prostate tissues of two individual mice and identified 200–1,200 base substitutions in each clone via WGS^88^. Using a few mutations shared by multiple clones, the researchers reconstructed the early phylogenies of the somatic cells. These efforts demonstrated that (1) the two cells of the two-cell stage mouse embryo do not contribute equally to adult cells and that (2) anatomically adjacent cells in adult tissue are not always developmentally closer than distant cells^88^. Most importantly, the work suggested that similar approaches may be applicable to human cells^63^ for understanding human embryogenesis, where the direct investigation is not possible.

#### Mutational burden of healthy and pathologic cells

Clonal organoids can be used to count somatic mutations accumulated in normal aged human cells (Fig. 3b). For example, Blokzijl and colleagues established clonal organoids from the human small intestine, colon, and liver tissues, followed by WGS^45^. The authors found that normal cells also accumulate a considerable number of somatic mutations as an individual ages. Given the observed mutational burden, these cells were estimated to endogenously accumulate ~40 base substitutions per year. The patterns of the mutations, or mutational signatures, suggested two intrinsic mechanisms (termed COSMIC SBS1 and SBS5^89^) underlying the age-related somatic mutations^90^.

Furthermore, the technique revealed the genomic differences of intestinal cells chronically exposed to pathologic environments. For example, gut epithelial cells in the colon of ulcerative colitis patients (chronic inflammatory bowel disease) were established as clonal organoids and sequenced^91^. Compared to control cells in healthy individuals, inflamed epithelial cells showed enriched somatic mutations in the genes of the IL-17 signaling pathway; this conclusion is supported by an independent study conducted through microdissection^92^.

The combination of clonal organoids and WGS has been employed to compare the mutational burden in normal and cancer cells in the same patient at an absolute single-cell resolution^93^. As expected, tumor cells carry several times more somatic mutations than normal colorectal cells; this is caused by additional mutational processes specifically present in neoplastic cells. More interestingly, tumor tissues harbor a very high level of intratumoral genome heterogeneity, with thousands of unique mutations in the single tumoral clones that are responsible for the clone-specific differences in responses to anticancer drugs. Although organoid-based techniques are being extensively applied to cancer tissues^94^, tumor organoids of some cancer types, such as lung adenocarcinomas, cannot yet be easily established^95^.

#### Genomic effects of specific genetic alterations

The possibility of long-term culture in organoids enables the investigation of the functional and mutational impacts of specific gene mutations in controlled experimental conditions (Fig. 3c). For example, to functionally validate the enrichment of clones with an impaired IL-17 pathway in the colorectal epithelium of ulcerative colitis patients^91,92,96^, the *NFKBIZ* gene was truncated by CRISPR–Cas9 editing in clonal colon organoids^91^. Indeed, the *NFKBIZ* mutant clones showed a higher proliferation rate than normal clones under culture conditions that mimicked the inflammatory tissue environment. This implies that clones with an impaired IL-17 pathway have a selective advantage in specific microenvironments.

Lynch syndrome is a representative hereditary cancer syndrome caused by inherited mutations in genes that affect DNA mismatch repair, including *MLH1*, *MSH2*, *MSH6*, and *PMS2*^97^^.^ Patients with Lynch syndrome frequently develop carcinomas of the colon, endometrium, stomach, and many other organs. To investigate the impact of the dysregulation of genes in nonneoplastic cells, Drost and colleagues performed *MLH1* knockout in colon organoids and cultured them to obtain multiple clonal organoids^87^. Two months of *MLH1*−/− organoid culture resulted in a significantly higher number of base substitutions and short indels, with characteristic mutational signatures in the specific sequence contexts.

#### Genomic impact of exogenous mutagens

The microbiota is associated with colon cancers^98,99^. Among the many bacterial species in the microbiota, the direct mutagenic effects of colibactin-producing *E. coli* were revealed by the exposure of organoids to the bacteria followed by clonalization and WGS^100^ (Fig. 3d). Colibactin increased the total number of somatic base substitutions and indels, which showed characteristic mutational patterns in the cocultured organoids. These signatures were validated in several cancer samples, including colorectal, head and neck, and urinary tract cancers, suggesting a mutagenic impact of the bacteria in these cancer types as well.

Cytotoxic chemotherapy is commonly used to treat metastatic cancers by inducing mutagenesis in cancer cells. The cytotoxic agents also affect normal cells. To investigate the genomic effects of one chemotherapeutic agent, 5-fluorouracil (5-FU), Christensen et al. treated normal intestinal organoids with 5-FU and then sequenced the organoids after clonalization^101^. Characteristic T > G substitutions in the CTT context (termed COSMIC SBS17^89^) were revealed in 5-FU-treated intestinal organoids, and they were also observed in 5-FU-treated cancers.

Similar approaches have been applied to reveal the mutagenic effects of ionizing radiation (IR) (Fig. 3d). IR is a strong carcinogen, but its characteristic mutational patterns have not been comprehensively identified^89^. Irradiated clonal organoids have a substantially higher mutational burden according to specific mutational signatures of short deletions and genomic rearrangements^102^.

#### Simultaneous multiomics profiling

In tumors, RNA expression and DNA methylation have been frequently investigated together with mutation identification^103,104^. More recently, the 3D genome organization has been correlated with genomic rearrangements^105^. By using the clonal organoid technique, such a multidimensional approach is possible in nonneoplastic normal cells (Fig. 3e). Notably, the integration of multiomics datasets can provide deep insights into the causative changes and treatment targets in human diseases^106^. However, primary tissues are not ideal for multiomics studies because (1) only a limited amount of material can be prepared, (2) intermixed signals are usually obtained from heterogeneous cell types, and (3) the long-term storage of the tissues is challenging. Organoids are more advantageous for this purpose because they provide pure signals from target cell types and can be readily cryopreserved. As organoid technology produces large numbers of progeny cells that are more than sufficient for WGS, a fraction of the cells can be reserved for further experiments after somatic mutation discovery. Once genome analyses reveal an interesting pattern, such as APOBEC-mediated hypermutations and/or complex genomic rearrangements, as observed under chromothripsis^107^, we can trace the origin and regulatory mechanisms of the mutational processes as well as their phenotypic consequences via clone-specific downstream analyses, such as transcriptome, epigenome and proteome profiling as well as experimental phenotyping. If necessary, more progeny cells can be easily produced via the further repopulation of the clonal line.

### Future directions

Genomics is a big data science and is becoming much larger in scale^108^. The first personal genome was reported in 2007^109^, and genome sequences from more than 500,000 human genomes had been produced as of 2016. It would not be very surprising, in fact, if the entire human population were to be sequenced in the next several decades. In parallel, an individual may be sequenced multiple times, at least for medical purposes, for instance, to longitudinally trace the genomic evolution of cancer tissues^110^ or to decompose the genomic heterogeneity of a specific individual^63,111^.

For the last decade, whole-exome and genome sequencing have been widely used for disease gene discovery studies, and many novel causal pathogenic genes of sporadic genetic diseases have been found^112^. However, success rates in ‘pinpointing’ causal mutations or mechanisms are estimated to be roughly 30−50%, even for clinically indisputable genetic disorders^113^. Indeed, there is a substantial level of ‘missing causal mutation’, which is not in accord with the current concept of the homogeneous genomes of individuals^15,114^.

The sequencing of single genomes provides the ultimate resolution in personal genomics. As an individual age, stochastically acquired genetic and epigenetic alterations make their somatic cells more heterogeneous, which is likely to lead to the generation and progression of human diseases. *In principle*, the formation, tissue distribution, and relative contribution of somatic mosaicism are stochastic personal events and can thus be variable across individuals. We first need to record the mutational and clonal complexity of human tissues on a massive scale to extract common features and principles. Technically, as mentioned throughout this manuscript, the landscape of somatic mosaicism is becoming fundamentally explorable, as seen in recent studies involving clones from diverse anatomical regions of human bodies^63,111^.

Importantly, cultural and ethical issues should be considered in this context. Methods of classical biology, such as the use of animal models, are not applicable here because it is not plausible to engineer hundreds of somatic mutations in even single cells, and the species-specific context between the endogenous mutation rate and lifespan cannot be fundamentally simulated in animal models. To reveal the direct association between single-cell mutations and human diseases and the trajectory of its longitudinal and spatial changes over time, a large number of human tissue specimens, not only from diseased but also from apparently healthy specimens, should be thoroughly investigated. To this end, social agreements and legal doctrines should be developed for donating precious diseased tissue (from both surgery and biopsy), organ transplant, and postmortem specimens for scientific research. Because genome sequencing involves individual privacy and confidentiality concerns, modalities for adequately protecting such information should be developed.

Although the mission is still ambitious, the technical advances mentioned above pave the way for the systematic decomposition of genomic heterogeneities in an individual at a single-cell resolution. The sensitive and precise detection of somatic mosaicism, not only in humans but also in many other model organisms, will be a major direction for future genetic studies and will be associated with genetic diseases.
